# Revisiting Goodenough-Kanamori rules in a new series of double perovskites LaSr_1−*x*_Ca_*x*_NiReO_6_

**DOI:** 10.1038/s41598-019-54427-0

**Published:** 2019-12-04

**Authors:** Somnath Jana, Payel Aich, P. Anil Kumar, O. K. Forslund, E. Nocerino, V. Pomjakushin, M. Månsson, Y. Sassa, Peter Svedlindh, Olof Karis, Vasudeva Siruguri, Sugata Ray

**Affiliations:** 1 Centre for Advanced Materials, Indian Association for the Cultivation of Science, Jadavpur, Kolkata 700032 India; 2 Department of Physics and Astronomy, Uppsala University, 752 36 Uppsala, Sweden; 3 School of Materials Science, Indian Association for the Cultivation of Science, Jadavpur, Kolkata 700032 India; 4 Department of Engineering Sciences, Uppsala University, 752 36 Uppsala, Sweden; 5 Department of Applied Physics, KTH Royal Institute of Technology, SE-164 40 Stockholm Kista, Sweden; 6 Laboratory for Neutron Scattering & Imaging, Paul Scherrer Institute, CH-5232 Villigen, PSI Switzerland; 7 UGC-DAE-Consortium for Scientific Research Mumbai Centre, 246C 2nd floor Common Facility Building (CFB), Bhabha Atomic Research Centre, Mumbai, 400085 India; 8 Present Address: Institute for Methods and Instrumentation in Synchrotron Radiation Research FG-ISRR, Helmholtz-Zentrum Berlin für Materialien und Energie, Albert-Einstein-Strasse 15, 12489 Berlin, Germany; 9 Present Address: Seagate Technology, 1 Disc Drive, Springtown, Northern Ireland BT48 0BF United Kingdom; 10 Department of Physics Chalmers University Of Technology, SE-412 96 Gteborg, Sweden

**Keywords:** Electronic properties and materials, Magnetic properties and materials

## Abstract

The magnetic ground states in highly ordered double perovskites LaSr_1−*x*_Ca_*x*_NiReO_6_ (*x* = 0.0, 0.5, 1.0) are studied in view of the Goodenough-Kanamori rules of superexchange interactions in this paper. In LaSrNiReO_6_, Ni and Re sublattices are found to exhibit curious magnetic states separately, but no long range magnetic ordering is achieved. The magnetic transition at ~255 K is identified with the independent Re sublattice magnetic ordering. Interestingly, the sublattice interactions are tuned by modifying the Ni-O-Re bond angles through Ca doping. Upon Ca doping, the Ni and Re sublattices start to display a ferrimagnetically ordered state at low temperature. The neutron powder diffraction data reveals long range ferrimagnetic ordering of the Ni and Re magnetic sublattices along the crystallographic *b-*axis. The transition temperature of the ferrimagnetic phase increases monotonically with increasing Ca concentration.

## Introduction

**Figure 1:**
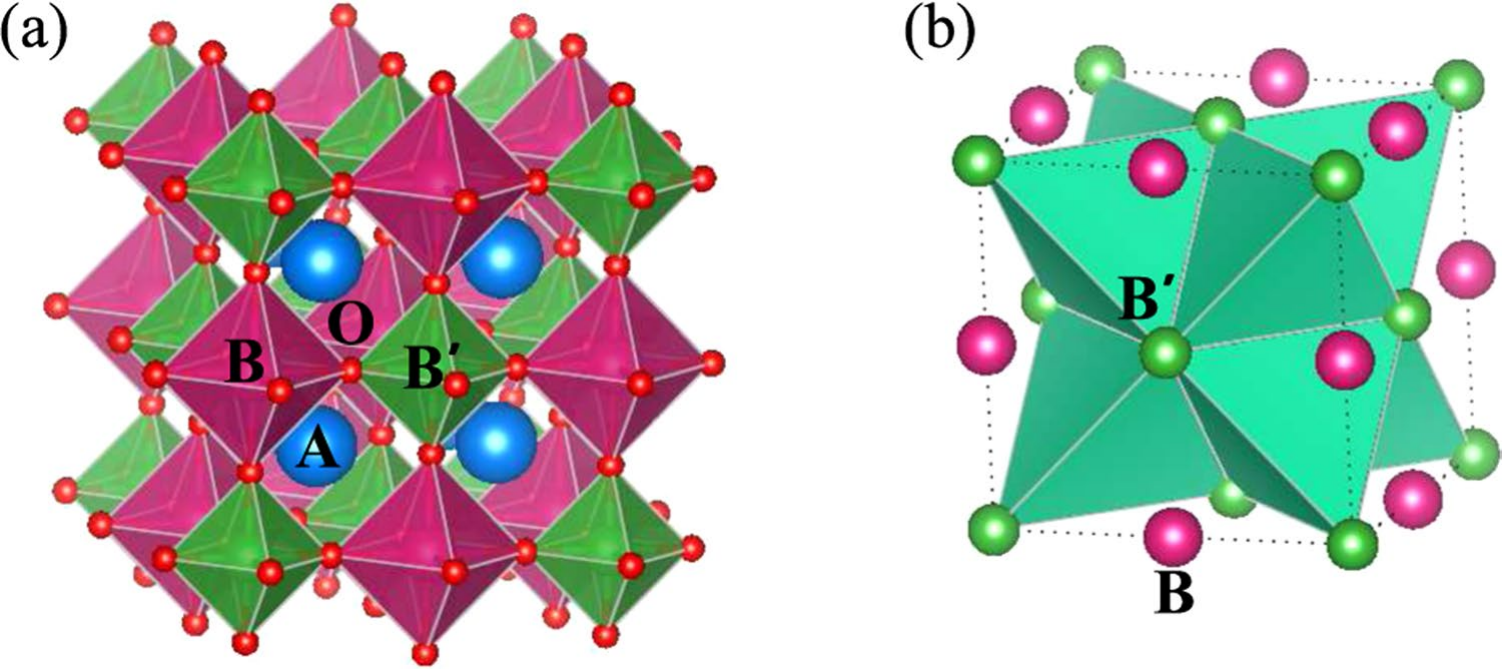
Crystal structure: (**a**) The crystal structure of the *B*-site ordered double perovskite, *A*_2_*BB*′O_6_. (**b**) The geometrically frustrated face-centered cubic lattice of edge-shared tetrahedra formed by the *B*′ sites.

Double perovskites (DP; *A*_2_*BB*′ O_6_)^1–3^ belong to a class of materials which exhibit many interesting properties and rich physics. Understandably, the choice of the transition metal ions at the *B* and *B*′ sites with different electron occupancies decide the material properties of the DPs. When both *B* and *B*′ are magnetic ions, the magnetic and electronic properties of the system are governed by *B*-O-*B*′ interaction within a rock salt type structural definition as shown in Fig. 1(a). For example, the high temperature ferromagnetic (FM) order (*T*_*C*_ > 400 K) of the DP compounds, Sr_2_FeMoO_6_ and Sr_2_FeReO_6_ is explained by a generalized double exchange mechanism through electronic band filling of the (Mo/Re) *t*_2*g*_↓-O-Fe *t*_2*g*_↓ conduction band^4^. However, if the *B*-site ion is non-magnetic, the magnetic ground state would be defined by the edge-shared network of tetrahedra comprising the *B*′ magnetic ions (Fig. 1(b)). Such systems often exhibit geometric frustration in presence of antiferromagnetic nearest-neighbor correlations. Recently, detailed theoretical investigations have been carried out on similar DPs with the magnetic *B*′ ions, having *nd*^1^ and *nd*^2^ (*n* = 4, 5) electronic configurations and significant spin orbit coupling (SOC)^5,6^. Here, the nearest neighbor distance between the tetrahedrally arranged 4*d*/5*d* magnetic ions naturally becomes much larger compared to the cases when both *B* and *B*′ sites are filled up with the magnetic ions. This reduces the inter-atomic exchange between the magnetic ions which helps to protect the SOC driven states. This situation opens up many options, and consequently many double perovskites with *d*^1^ (e.g., Ba_2_YMoO_6_, Sr_2_CaReO_6_, Sr_2_MgReO_6_, Ba_2_NaOsO_6_ etc.) as well as *d*^2^ electronic configurations (e.g., Ba_2_CaOsO_6_, Ba_2_YReO_6_, La_2_LiReO_6_ etc.) have been studied, where numerous unusual magnetic ground states are revealed^7–15^.

Another interesting possibility appears in DPs, when both *B* and *B*′ ions are magnetic, but the valence electrons of *B* ion lack the orbital symmetry of the same of *B*′ ion for effective *B*-O-*B*′ super-exchange interaction. Such a situation will give rise to two noninteracting or weakly interacting magnetic sublattices. It will be interesting to gradually manipulate the extent of *B*-O-*B*′ interaction by carefully changing the *B*-O-*B*′ angle, and consequently follow the evolution of two sublattices getting engaged in single magnetic lattice (Fig. 1(b) → Fig. 1(a)), following the famous Goodenough-Kanamori rule. Accordingly, we have designed a series of DPs, LaSr_1−*x*_Ca_*x*_NiReO_6_, having a combination of 3*d* and 5*d* transition metals Ni^2+^ (3*d*^8^, *t*_2*g*_^6^*e*_*g*_^2^) and Re^5+^ (5*d*^2^, *t*_2*g*_^2^) at the *B* and *B*′ sites, respectively. Due to large crystal field splitting, the empty *e*_*g*_ orbitals of Re have much higher energy than the *t*_2*g*_ manifold and do not contribute in any interatomic exchange interaction. The filled *t*_2*g*_ orbital of Ni as well has less involvement in the exchange interaction. The only possible exchange interaction that can be active between the *e*_*g*_ of Ni^2+^ and the *t*_2*g*_ of Re^5+^, will be very weak if the *B*-O-*B*′ angle is strictly 180°, as the overlap integral between the *e*_*g*_ and the *t*_2*g*_ becomes zero. However, finite overlap between these orbitals can be introduced by tuning the bond angles and lattice parameters as a consequence of the doping of Sr^2+^ ions by smaller sized Ca^2+^ ions.

In our design criteria of the ordered LaSr_1−*x*_Ca_*x*_NiReO_6_ series, *B*, *B*′ cations are also selected in such a way that there are large differences in their charge and ionic radii, in order to achieve complete *B*, *B*′ rock-salt ordering. Here the ionic radii of Ni^2+^ (=0.69 Å) and Re^5+^ (=0.58 Å)^16^ do fulfill the above criteria. Detailed magnetic measurements on LaSr_1−*x*_Ca_*x*_NiReO_6_ indicate curious evolution of magnetic states as a function of doping. For LaSrNiReO_6_, the system undergoes multiple magnetic transitions, indicated by a divergence between ZFC and FC at ~255 K, typical of Re^5+^ *t*_2*g*_^2^ ions confined in a fcc sublattice, and by a down turn in magnetization at ~27 K, observed in both the ZFC and FC curves. Transport measurements confirm purely insulating behavior of the samples. However with Ca doping, the structure undergoes into larger monoclinic distortion, which results in a larger deviation of the Ni-O-Re (∠NOR) bond angles from 180°. This deviation enables substantial superexchange interaction between the Ni *e*_*g*_ and Re *t*_2*g*_ orbitals, resulting to an overall ferrimagnetic order between two individual ferromagnetic sublattices.

## Results

**Figure 2:**
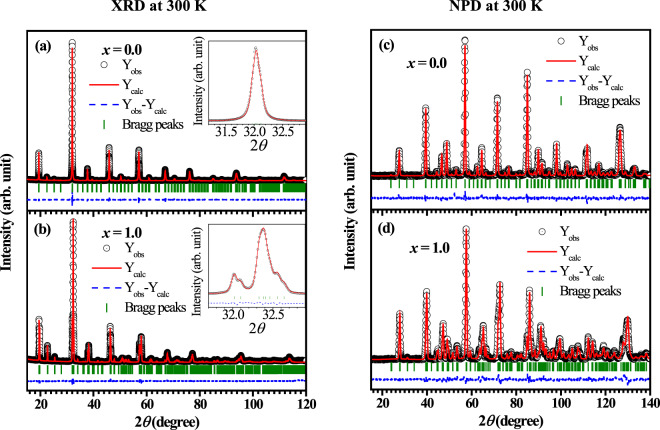
Combined Rietveld refinement of the X-ray diffraction pattern and the neutron diffraction pattern collected at room temperature: The observed (black circle), calculated (red line) and the difference (blue dashed line) diffraction data for (**a**,**c**) *x* = 0.0, (**b**,**d**) *x* = 1.0 compositions are shown. Insets of (**a**,**b**) show the magnified view of the peaks around 2*θ* ~ 32°, emphasising the monoclinic distortion in case of *x* = 1.0 sample.

All the samples appeared to be single phased as no impurity peak is detected in the whole 2*θ* range in the powder XRD data, collected at room temperature for LaSr_1−*x*_Ca_*x*_NiReO_6_ (LSCNRO) series (see Fig. 2(a,b)). The observed (circle), calculated (line through the data) and the difference (blue dashed line) diffraction data are shown in Fig. 2 for *x* = 0.0 and 1.0 compositions respectively, which could be fitted with the *P*2_1_/*n* space group. The system shows increase in monoclinic distortion (see the insets to Fig. 2(a,b)) with increasing Ca concentration (see Fig. S1 in SI to compare the XRD patterns for all the three compositions collected at a synchrotron X-ray source). The observation of the monoclinic distortion in this series of compounds is in agreement with the calculated tolerance factors (*f*), which goes from *f* = 0.98 (*x* = 0.0) to *f* = 0.96 (*x* = 1.0). An overall shift of the Bragg peaks towards higher 2*θ* indicates a decrease of the unit cell parameters with increasing *x*. Presence of the intense peak at around 19.6° (1 1 0) indicates high degree of Ni/Re ordering within the double perovskite structure of LaSr_1−*x*_Ca_*x*_NiReO_6_ for *x* = 0.0 and 1.0 compositions. A combined refinement of XRD and NPD patterns was carried out for both the compositions at 300 K in order to accurately quantify Ni and Re cation ordering and determine the oxygen positions. The refined structural parameters along with the bond lengths and bond angles are given in Tables 1 and 2 respectively. The ordering between Ni and Re is quantified to be 96% and 99% for *x* = 0.0 and *x* = 1.0 from the refined occupation numbers of Ni1/Re1 and Re2/Ni2 at (0.5, 0.0, 0.5) and (0.5, 0.0, 0.0) crystallographic sites, respectively. The stoichiometry of Ni and Re in the compounds are probed and confirmed through ICP-OES experiments (see SI). The Bond Valence Sum (BVS) for Ni is calculated from the Ni-O bond lengths listed in Table 2^17^. The values obtained are 2.2 for both the composition which is in accordance with the expected oxidation state of the Ni ions in these compounds.

**Table 1:** Refinement parameters of the *x* = 0.0 and 1.0 compositions obtained from combination of the XRD and NPD data collected at 300 K and NPD data at 2 K.

Space group	*x* = 0.0		*x* = 1.0	
	300 K	2 K	300 K	2 K
	*P*2_1_/*n*	*P*2_1_/*n*	*P*2_1_/*n*	*P*2_1_/*n*
*a* (Å)	5.5980 (1)	5.5862 (1)	5.49735 (5)	5.48136 (8)
*b* (Å)	5.5748 (1)	5.5693 (1)	5.58551 (4)	5.59108 (8)
*c* (Å)	7.8947 (2)	7.8846 (1)	7.80465 (7)	7.78278 (9)
*β*	90.021 (8)	90.045 (2)	89.8729 (9)	89.924 (4)
Cell Vol. (Å^3^)	246.38 (1)	245.30 (1)	239.638 (3)	238.517 (6)
R _*wp*_	5.21	4.58	4.39	6.81
*χ*^2^	2.17	4.98	6.37	1.99
**La/(Sr/Ca)**				
*x*	0.4896 (3)	0.4977 (7)	0.5015 (4)	0.4874 (6)
*y*	0.5225 (1)	0.5251 (3)	0.5381 (1)	0.5404 (3)
*z*	0.2461 (2)	0.2525 (9)	0.2513 (1)	0.2503 (9)
B (Å^2^)	0.57 (2)	0.48 (4)	0.56 (5)	0.51 (4)
**Ni1/Re1**				
occupancy	0.96/0.04	0.96/0.04	0.99/0.01	0.99/0.01
B (Å^2^)	0.42 (1)	0.40 (4)	0.48 (1)	0.45 (4)
**Re2/Ni2**				
occupancy	0.96/0.04	0.96/0.04	0.99/0.01	0.99/0.01
B (Å^2^)	0.42 (1)	0.40 (4)	0.48 (1)	0.45 (4)
**O1**				
*x*	0.294 (1)	0.291 (1)	0.2958 (9)	0.2916 (9)
*y*	0.289 (1)	0.287 (1)	0.2923 (9)	0.2827 (9)
*z*	0.034 (1)	0.035 (1)	0.0472 (9)	0.0510 (7)
B (Å^2^)	0.72 (4)	0.52 (1)	0.89 (5)	0.71 (5)
**O2**				
*x*	0.237 (1)	0.228 (1)	0.2111 (9)	0.2023 (9)
*y*	0.769 (1)	0.767 (1)	0.7937 (9)	0.8025 (9)
*z*	0.024 (1)	0.025 (1)	0.0349 (9)	0.0378 (7)
B (Å^2^)	0.73 (5)	0.69 (4)	0.79 (9)	0.70 (5)
**O3**				
*x*	0.573 (1)	0.571 (1)	0.5856 (7)	0.5821 (5)
*y*	−0.0014 (9)	−0.0015 (9)	−0.0217 (6)	−0.0220 (4)
*z*	0.247 (2)	0.248 (1)	0.2481 (9)	0.2413 (9)
B (Å^2^)	0.68 (2)	0.61 (3)	0.80 (5)	0.78 (1)

The *x*, *y*, *z* coordinates for Ni1/Re1 and Re2/Ni2 are 1/2,0,1/2 and 1/2,0,0 respectively.

**Table 2:** (Ni,Re)-O bond distances and Ni-O-Re bond angles extracted from the data of Table 1.

	*x* = 0.0		*x* = 1.0	
	300 K	2 K	300 K	2 K
**Bond length/Å**				
Ni1-O1	2.041 (9)	2.032 (6)	2.032 (6)	2.047 (7)
Ni1-O2	2.014 (9)	1.978(1)	2.029 (6)	2.045 (7)
Ni1-O3	2.04 (1)	2.02 (9)	2.023 (8)	2.066 (8)
Re2-O1	2.000 (9)	1.998 (9)	2.015 (6)	1.990 (7)
Re2-O2	1.964 (9)	2.00 (4)	1.980 (6)	1.991 (8)
Re2-O3	1.99 (1)	1.99 (5)	1.998 (8)	1.936 (8)
**Angle/°**				
Ni1-O1-Re2	155.6 (5)	156.1(3)	151.1 (4)	151.7 (4)
Ni1-O2-Re2	166.6 (5)	165.2 (3)	155.6 (4)	151.9 (4)
Ni1-O3-Re2	156.5 (4)	157.1 (4)	152.0 (3)	152.9 (1)

**Figure 3:**
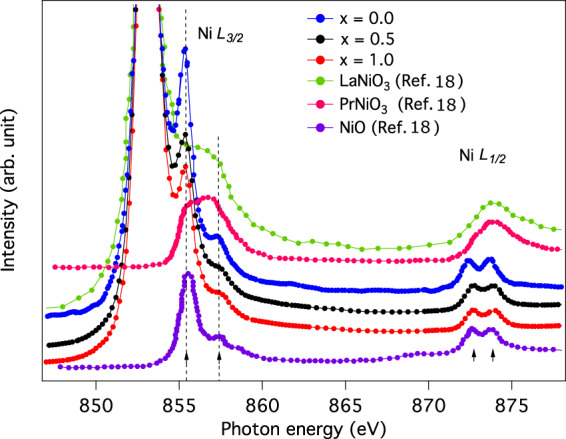
X-ray absorbtion spectra (XAS): Ni *L*-edge XAS measured at room temperature for *x* = 0.0, 0.5 and 1.0 compositions of LaSr_1−*x*_Ca_*x*_NiReO_6_ series are plotted together with the reference spectra of Ni XAS from LaNiO_3_, PrNiO_3_ and NiO samples. The reference spectra are plotted after digitizing and rescaling the data points from the Figs. 4 and 6 of the ref. ^18^. Data have been vertically shifted for clarity.

**Figure 4:**
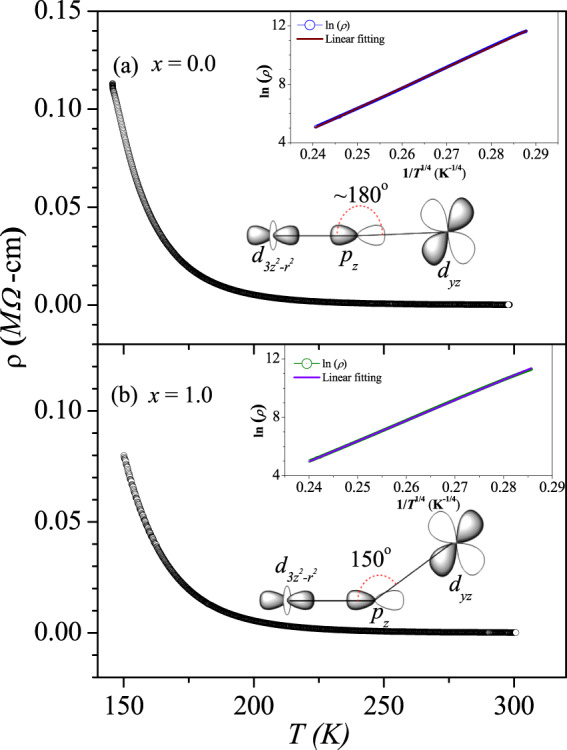
Resistivity vs temperature measurements: Resistivity vs temperature plots for (**a**) *x* = 0.0 and (**b**) *x* = 1.0 compositions. Insets showing the insulating electrical resistivity of both the compositions could be modelled by variable range hopping mechanism in 3-dimension.

Next, we have performed X-ray absorption spectroscopy (XAS) at the *L*-edge of Ni to further verify the charge state. The XAS spectra collected for *x* = 0.0, 0.5, 1.0 compositions are shown in Fig. 3, together with the reference spectra for Ni^2+^ and Ni^3+^. The reference spectra are obtained by digitizing and recalling the data points from the Figs. 4 and 6(a) of the ref. ^18^. Note that the La *M*_3/2_ absorption edge is superimposed with the Ni *L*_3/2_-edge. The spectral features of both the Ni *L*_3/2_ and *L*_1/2_ are quite different for Ni^2+^ and Ni^3+^. Both *L*_3/2_ and *L*_1/2_ consist of two peaks indicated by the arrows in the figure. In the 2+ charge state of Ni as in NiO, the lower energy peak of *L*_3/2_ is much more intense relative to the higher energy one, while both the peaks have similar intensities in case of NdNiO_3_, PrNiO_3_ and LaNiO_3_, containing Ni^3+^ ions^18^. Also, the spectral feature of the *L*_1/2_ edge of all the three compositions, clearly resemble to that of NiO. Hence it can be inferred that Ni is in 2+ charge state for all the LSCNRO compositions. In order to maintain charge neutrality, the charge state of Re should then be 5+ as Sr^2+^, La^3+^ and O^2−^ are expected to be very stable at their respective ionic states.

The electrical resistivity (*ρ*) of the two end member compounds as a function of temperature are shown in Fig. 4. The resistivity data were fitted using an activated transport model as well as using variable range hopping model. Both the data were nonlinear on a *T*^−1^ scale and was found to be linear on a *T*^−1/4^ scale (see inset to Fig. 4(a,b)), in accordance with a three-dimensional variable range hopping transport model^19^. The insulating nature of the compounds can be explained by the fact that Ni^2+^; 3*d*^8^ effectively provides electrons of *e*_*g*_ symmetry at the valence band as the *t*_2*g*_ bands are completely filled up, while Re^5+^; 5*d*^2^ has partially filled *t*_2*g*_ bands, thus from the symmetry consideration the electron hopping is prohibited (see the inset of Fig. 4(a)). However, a nonzero hopping probability is realized if the bond angles deviate sufficiently from 180°, thereby enabling Ni*e*_*g*_-Re *t*_2*g*_ hybridization via oxygen (compare the inset to Fig. 4(b)). Replacing Sr^2+^ by the smaller Ca^2+^ ion, we anticipate a larger octahedral distortion that can provide a route for hybridization between the Ni*e*_*g*_ and Re *t*_2*g*_. Indeed a larger amount of distortion is achieved as understood from the crystal structure of LaCaNiReO_6_ and consequently a clear decrease in the resistivity is also observed, although the temperature dependence still suggests that the material is best described as an insulator/semiconductor.

**Figure 5:**
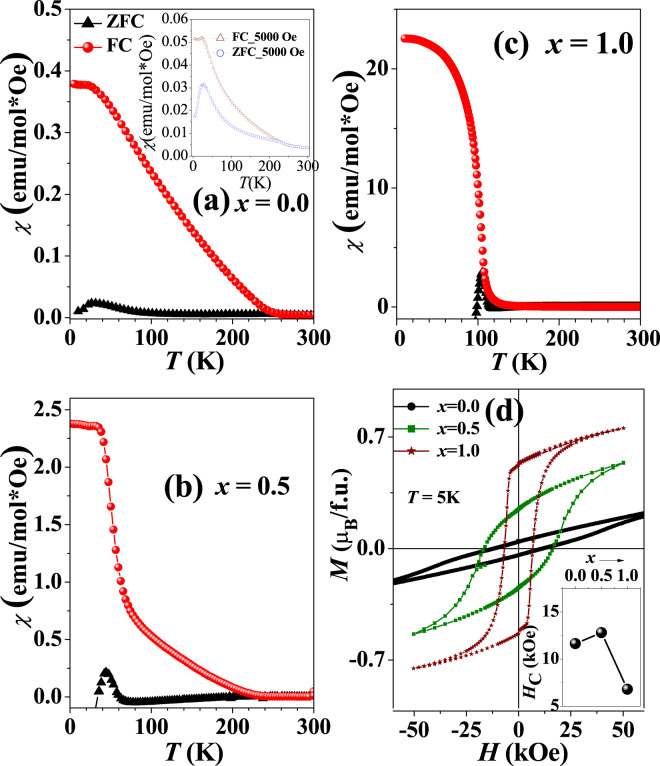
Magnetic measurements: (**a**–**c**) ZFC FC, *M***(*T*)** data of LaSr_1−*x*_Ca_*x*_NiReO_6_ with *x* = 0.0, 0.5 and 1.0 measured with *H* = 200 Oe. Inset of (**a**) shows the temperature dependence of magnetic susceptibility of *x* = 0.0 composition in an applied field of 5000 Oe. (**d**) *M***(*H*)** curves for *x* = 0.0, 0.5 and 1.0 compositions. Inset shows variation of *H*_*C*_ with doping.

Next, we have looked into the magnetic properties in details. The zero field cooled (ZFC) and field cooled (FC) data recorded with an applied field of 200 Oe for the three compositions of LSCNRO are shown in Fig. 5(a–c). The *M*(*T*) of *x* = 0.0 sample shows a transition at around 255 K where ZFC and FC curves start to bifurcate. Another transition occurs at around 27 K, where susceptibility seems to saturate. In the high temperature regime (255–300 K), the inverse susceptibility follows the Curie-Weiss behavior resulting (see Fig. S2(a)) in a effective moment of *μ*_*eff*_ = 3.07 *μ*_*B*_. The observed magnetic moment is lower than the expected spin-only contribution of both Ni^2+^ and Re^5+^ and this reduction in magnitude can attributed to the spin-orbit coupling in Re^4,20^. For *x* = 0.5 (Fig. 5(b)) a ferro/ferrimagnetic (FM) like transition is observed at 45 K, along with the high temperature FC-ZFC bifurcated curves. This FM like transition gradually shifts to higher temperatures with increasing *x* (~110 K for *x* = 1.0; Fig. 5(c)). Also as *x* increases, the FM like transition becomes stronger to obscure the high temperature FC-ZFC splitting. The effective magnetic moment obtained from Curie-Weiss fit for *x* = 1.0 (see Fig. S2(b)) is 3.59 *μ*_*B*_.

The magnetization versus field (*M*(*H*)) data, collected at 5 K for the three compositions are plotted in Fig. 5(d). The coercivity of all the samples are very high in general, which arises as a result of large spin-orbit coupling driven anisotropy, commonly observed for Re based DPs^4,20^. However, the overall nature of the *M*(*H*) curves varies drastically with *x*. The remnant magnetization increases continuously with clear signatures of magnetic saturation as a function of Ca-doping. This observation clearly suggests significant changes in magnetic interactions with increasing monoclinic distortions and decreasing *B*-O-*B*′ angle.

**Figure 6:**
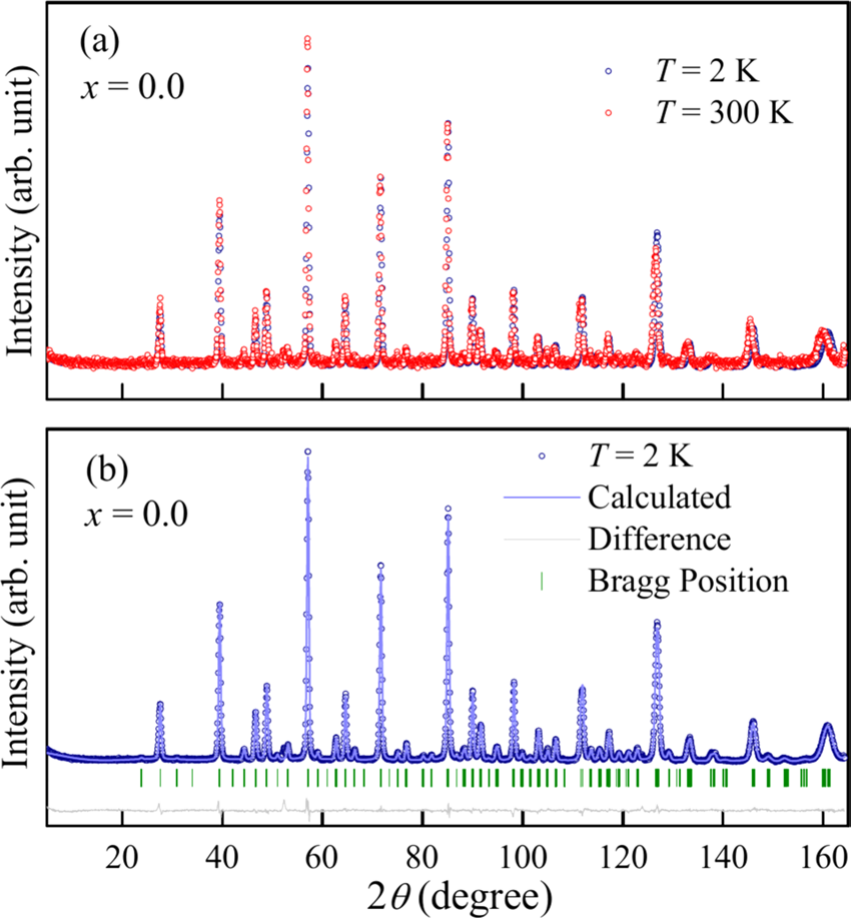
Neutron diffraction measurements for ***x*** = 0.0 composition: (**a**) Neutron diffraction pattern for *x* = 0.0, recorded at 2 K and 300 K. No magnetic Bragg peaks are observed. (**b**) Rietveld refinement of NPD data at 2 K. The solid line through the experimental points is the Rietveld refinement profile calculated for the space group *P*2_1_/*n* structural model. The vertical bars indicate the Bragg positions. The lowermost curve represents the difference between the experimental data and the calculated results.

In order to have more insight about the observed magnetic transitions, neutron powder diffraction (NPD) were carried out for the end compositions at 2 K. The changes in the lattice parameters, bond lengths and bond angles with temperature have been listed in Tables 1 and 2. For *x* = 0.0, there is a contraction of the lattice parameters with decrease in temperature with no signature of long range magnetic ordering (see Fig. 6(a)), which suggests that the observed magnetic transitions in the magnetization curves are of short range type. Our observation is in accordance with the NPD measurements of SrLaNiReO_6_ reported in ref. ^21^ which also lacked evidence of long-range magnetic ordering.

**Figure 7:**
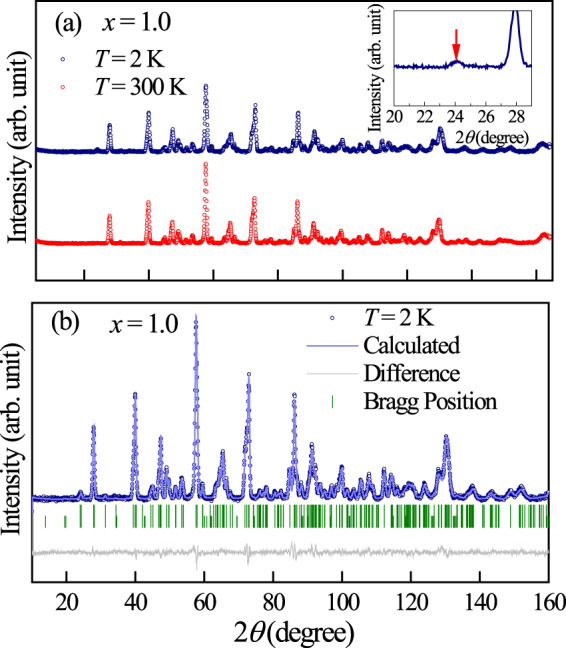
Neutron diffraction measurements for ***x*** = 1.0 composition: (**a**) Neutron diffraction pattern for *x* = 1.0 recorded at 2 K and 300 K. Inset shows the enhanced intensity of the nuclear Bragg peak at 24° at 2 K. Rietveld refinement of NPD data for *x* = 1.0 at (**b**) *T* = 2 K (considering magnetic structure). The solid line through the experimental points is the Rietveld refinement profile calculated for the space group *P*2_1_/*n* structural model. The vertical bars indicate the Bragg positions. The lowermost curve represents the difference between the experimental data and calculated results.

**Figure 8:**
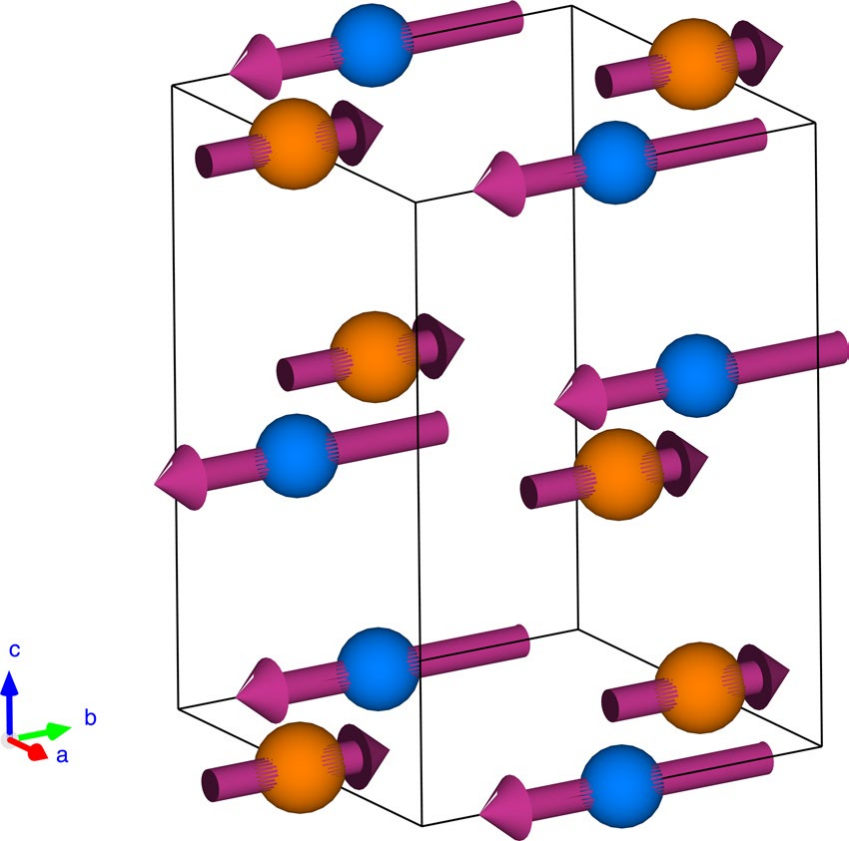
Magnetic structure for ***x*** = 1.0 at ***T*** = 2 K: Arrows indicate the direction of the Ni (blue sphere) and Re (orange sphere) moments. The La/Ca, and O atoms are not shown for clarity. The average value of the ordered magnetic moment is estimated as ~1.0 *μ*_*B*_ /f.u.

The neutron diffraction patterns of *x* = 1.0 recorded at 2 K and 300 K are shown in Fig. 7(a). From, Table 1 it is evident that there is a elongation in the lattice parameter along *b* axis with the decrease in temperature. Contrary to *x* = 0.0, it is observed that the nuclear peak profile in the vicinity of 24° comprising(011), (101), and (−101) nuclear Bragg peaks clearly gets enhanced in intensity at 2 K. Since no extra magnetic reflections with significant intensity are observed, the magnetic structure can be assumed to coincide with the crystal structure and propagation vector ***k*** = (000) may be used to model the structure. Thus, using the formalism of propagation vectors in conjunction with the irreducible representation analysis as described in ref. ^22^, the magnetic reducible representation for the magnetic atoms can be decomposed as a direct sum of irreducible representations. For the refinement of the magnetic structure, the symmetry elements and basis vectors of the irreducible representations (Γ) for magnetic Ni (Re) atom were obtained using *BasIre*p*s* program available within the FullProf suite, using the propagation vector ***k*** = (000). It is observed that the magnetic representation of Ni (Re) ion comprises two irreducible representations Γ = Γ_1_ + Γ_3_. The two Γ values were tested sequentially with the measured pattern at 2 K and we could refine the magnetic structure satisfactorily using the basis vectors of Γ_1_. Although using this same representation, a collection of peaks at around 2*θ* = 47° shows magnetic contributions, the magnetic structure factor turns out to be less than 1% of the nuclear intensity with magnetic form factor for Ni^2^^+^ being around 0.7 at this Q-value, which hinders the accurate estimation of the magnetic structure from this peak. Thus the most probable magnetic structure can be estimated from the peak near 24° using the basis vectors of Γ_1_. It was found that the Ni (Re) moments lie predominantly along negative (positive) *b* direction with a very small canting, which could not be resolved within the measuring capability. Hence, the moments were constrained along *b* direction and refined. The obtained moments on Ni and Re are −2.0 (4) *μ*_*B*_ and 1.0 (4) *μ*_*B*_ (Table 3, Fig. 8), respectively, resulting in a net magnetic moment of ~1.0 *μ*_*B*_ per formula unit along the negative *b* direction.

**Table 3:** Results obtained from NPD data collected at 2 K for *x* = 1.0 sample.

*x* = 1.0 (LaCaNiReO_6_)	
Atom type	*M*_*tot*_ (*μ*_*B*_)
Ni	−2.0 (4)
Re	1.0 (4)

*M*_*y*_ is refined and the net magnetic moment is listed above.

## Discussions

In a highly ordered *B*-site double perovskite with magnetic ions at *B* and *B*′ sites the long range magnetic order is always determined by the type of active exchange interaction that mediates through the *B*-O-*B*′ connectivity. For localized electrons (i.e. the *d* electrons), the magnetic interaction between two transition metal *B* and *B*′ cations is often described by the Goodenough-Kanamori rules of superexchange interaction^23,24^. According to this rule, when the orbitals of two magnetic ions have a significant overlap integral, the superexchange is antiferromagnetic (∠*e*_*g*_(*B*)-O-*e*_*g*_(*B*′) = 180°, ∠*t*_2*g*_(*B*)-O-*t*_2*g*_(*B*′) = 180°, ∠*t*_2*g*_(*B*)-O-*e*_*g*_(*B*′) = 90°). However, when the orbitals are arranged in such a way that they are expected to be in contact but to have no/weak overlap integral - most notably *t*_2*g*_ and *e*_*g*_ in 180° position, where the overlap is zero by symmetry, the rules predict ferromagnetic interaction, which is usually very weak in strength. In case of highly ordered LaSr_1−*x*_Ca_*x*_NiReO_6_ compounds, Ni 3*d* and Re 5*d* orbitals are connected via oxygen 2 *p* orbitals. From refinement of XRD and NPD, the average Ni-O-Re bond angle (∠NOR) comes about 160° for *x* = 0.0 sample and ~152° for *x* = 1.0 sample. Therefore in case of *x* = 0.0 sample, when ∠NOR is closer to 180°, the superexchange interaction between half filled *e*_*g*_ orbitals of Ni^2+^ and partially filled *t*_2*g*_ orbitals of Re^5+^ is a weak ferromagnetic interaction. Of course, there could be magnetic signals appearing from independent Ni and Re sublattices as well. However, as the ∠NOR start deviating noticeably from 180° (e.g. *x* = 1.0), a sizeable overlap between the *e*_*g*_ and *t*_2*g*_ orbitals starts to favor antiferromagnetic (AFM) interaction between the partially filled Ni *e*_*g*_ and Re *t*_2*g*_ orbitals following the Goodenough-Kanamori rule. Further, we have noticed that the smaller volume of the *x* = 1.0 sample compared to *x* = 0.0 sample has no significant effect in the Ni/Re-O bond lengths. The reduction of the lattice parameters are compensated by the smaller ∠NOR angles.

We conclude that the low temperature magnetic feature observed in *x* = 0.0 compound could very well be a reminiscence of the weak ferromagnetism predicted by Goodenough-Kanamori rule, but could also be an independent Ni sublattice feature as is seen in other Ni analog samples, such as, Sr_2_NiWO_6_ and Sr_2_NiTeO_6_ with nonmagnetic W^6+^ ([*Xe*] 4*f* ^14^) and Te^6+^ ([*Kr*] 4*d*^10^) at the *B*′ site, where the antiferromagnetic transition occurs at 35 K and 54 K, respectively^25^. This low temperature transition has been identified as the onset of a spin glass behavior in *x* = 0.0 compound^21^. On the other hand, the observed bifurcation between ZFC and FC curves at higher temperatures in *x* = 0.0 compound is clearly very similar to what is commonly observed when Re-ion sits in the geometrically frustrated fcc lattice (the *B*′-site) within the double perovskite structure, but having nonmagnetic *B*-site ions, e.g., Sr_2_CaReO_6_^10^, Sr_2_InReO_6_^26^.

For *x* = 1.0 sample, further deviation of ∠NOR from 180° compared to that of *x* = 0.0 enables an antiferromagnetic interaction between Ni^2+^ and Re^5+^ ions. In an ordered structure, this will result the moment of the individual sublattice to order along the same direction. Strong spin-orbit interaction may result magneto-crystalline anisotropy in case of the Re 5*d* spins, while the Ni spins, residing in more isotropic 3*d* orbitals, will orient antiparallel to Re spins following the super-exchange interaction. Therefore, the final magnetic structure is resulted in an ferrimagnetic arrangement between the two sublattices along the crystallographic *b*-axis. In LSCNRO, both Ni^2+ ^and Re^5+ ^ions have two unpaired electrons. However, the strong spin-orbit coupling in 5*d* orbitals (relative to 3*d* orbitals) usually results in a reduced total moment in Re ions compared to its spin only value^27–29^, which is clearly observed from the NPD analysis. The net magnetic moment of ~1.0 *μ*_*B*_ /f.u. obtained from NPD is in good agreement with the observed moment in *M* vs *H* data at the highest applied field ~0.75 *μ*_*B*_ /f.u.).

In conclusion, a series of double perovskites, LaSr_1−*x*_Ca_*x*_NiReO_6_ (*x* = 0.0, 0.5, 1.0) is realized with partially filled orbitals of *e*_*g*_ and *t*_2*g*_ symmetries (local) at highly ordered *B* and *B*′-sites respectively. All the compositions adopt a monoclinic structure. In LaSrNiReO_6_ (*x* = 0.0), an unusual divergence between the ZFC and FC curves is identified with the magnetic state that arises from Re *t*_2*g*_^2^ ions sitting in a geometrically frustrated fcc sublattice in DP host. At low temperature (~27 K), the system undergoes another magnetic transition, where weak ferromagnetism predicted by Goodenough-Kanamori rule could be identified. As lattice parameter decreases with Ca doping, the reduced Ni-O-Re bond angle introduces nonzero overlap integral between Ni *e*_*g*_ and Re *t*_2*g*_ orbitals, which favors a ferrimagnetic alignment between Ni and Re sublattices along the *b*-axis. The neutron powder diffraction measurement conducted at room temperature and low temperature (2 K) revealed that the *x* = 0.0 sample possesses a disordered/short-range magnetic state at low temperature, while for Ca sample, the Ni and Re sublattices aligned ferrimagnetically to give a long range magnetic order evidenced from the magnetic Bragg peak corresponding to the double perovskite superlattice peak.

## Methods

Four samples of LaSr_1−*x*_Ca_*x*_NiReO_6_ (LSCNRO) (*x* = 0.0, 0.5, 1.0) were synthesized by solid state synthesis route. Highly pure La_2_O_3_, SrCO_3_, CaCO_3_, NiO, Re_2_O_7_ and Re metal were used as the starting materials. The synthesis was done in two steps. In the first step, La_2_NiO_4_ was made by heating a mixture of stoichiometric La_2_O_3_ and NiO at 1250 °C in an inert atmosphere for 48 hours with several intermediate grinding. SrO and CaO were used after heating them at 1250 °C and 1000 °C for 12 hours in an inert atmosphere. Next, stoichiometric amount of La_2_NiO_4_, SrO, CaO, NiO, Re_2_O_7_ and Re metal were mixed inside a glovebox and the resultant mixture was sealed inside a quartz tube, which was then annealed at 1200 °C for the final product.

The phase purity of the three samples (*x* = 0.0, 0.5, 1.0) were checked by x-ray diffraction (XRD) Bruker AXS: D8 Advance x-ray diffractometer (Cu *K*_*α*_; *λ*_*α*_ = 1.54059 Å) as well as at MCX beamline of the Elettra Synchrotron Centre, Italy using wavelength of 0.751 Å. The XRD data were analyzed via Rietveld refinement using the FullProf ^30^ program. La, Ca, Sr, Ni, Re quantitative analysis were performed in Inductively Coupled Plasma Optical Emission Spectroscopy (ICP-OES) (Perkin-Elmer USA, Optima 2100 DV) instrument following standard protocol of sample analysis. *d*.*c*. magnetic measurements were carried out in a Quantum Design SQUID magnetometer. Resistivity measurements were performed in a home made four probe setup. Soft x-ray absorption spectroscopy (XAS) was performed at I1011 beamlines of the Swedish synchrotron facility MAX-lab, Lund. All the XAS spectra were measured by recording the total electron yield. Neutron powder diffraction (NPD) measurements were performed using the HRPT^31^ diffractometer at the Paul Scherrer Institut, SINQ (Switzerland). The neutron wavelength was set to *λ* = 1.89 Å and about 1 g of *x* = 0.0 and *x* = 1.0 samples were used. Magnetic structure refinements were performed using the FullProf suite^30^.

## Supplementary information

Supplementary information
