# Supplementary material for: Revisiting Goodenough-Kanamori rules in a new series of double perovskites LaSr1−xCaxNiReO6
Source: Sci Rep. 2019 Dec 4;9:18296. doi: 10.1038/s41598-019-54427-0 (PMC6892940; doi:10.1038/s41598-019-54427-0)
Supplement: Supplementary file 1 — Supplementary information [file 41598_2019_54427_MOESM1_ESM.docx]

**Revisiting Goodenough-Kanamori rules in a new series of double**

**perovskites LaSr_1-_*_x_*Ca*_x_*NiReO_6_**

Somnath Jana,1, 2, ∗ Payel Aich,3 P. Anil Kumar,4, † O. K. Forslund,5 E. Nocerino,5 V. Pomjakushin,6 M. Månsson,5 Y. Sassa,2 Peter Svedlindh,4 Olof Karis,2 Vasudeva Siruguri,7 and Sugata Ray1, 3

1Centre for Advanced Materials, Indian Association for the Cultivation of Science, Jadavpur, Kolkata 700032, India

2Department of Physics and Astronomy, Uppsala University, 752 36 Uppsala, Sweden

3School of Materials Science, Indian Association for the Cultivation of Science, Jadavpur, Kolkata 700032, India

4Department of Engineering Sciences, Uppsala University, 752 36 Uppsala, Sweden

5Department of Applied Physics, KTH Royal Institute of Technology, SE-164 40 Stockholm Kista, Sweden

6Laboratory for Neutron Scattering & Imaging, Paul Scherrer Institute, CH-5232 Villigen PSI, Switzerland

7UGC-DAE-Consortium for Scientific Research Mumbai Centre, 246C 2^nd^ floor Common Facility Building (CFB), Bhabha Atomic Research Centre, Mumbai 400085, India

∗ Corresponding author: sj.phys@gmail.com; Present Address: Institute for Methods and Instrumentation in Synchrotron Radiation Research FG-ISRR, Helmholtz-Zentrum Berlin für Materialien und Energie, Albert-Einstein-Straße 15, 12489 Berlin, Germany

† Present Address: Seagate Technology, 1 Disc Drive, Springtown, Northern Ireland BT48 0BF, United Kingdom

**Figure S1: Rietveld refinement of the X-ray diffraction pattern collected at room temperature:** The data were collected at MCX beamline of the Elettra Synchrotron Centre, Italy using wavelength of 0.751 Å. The observed (black circle), calculated (red line) and the difference (blue dashed line) diffraction data for (a) *x* = 0.0, (b) *x* = 0.5, (c) *x* = 1.0 compositions are displaced. Insets show the magnified view of the peaks around 2𝜃 ∼ 15°-16^o^, emphasizing the monoclinic distortion in case of *x* = 1.0 sample.

**Table S1: Compositional Study through ICP-OES**

| Sample | La | Sr/Ca | Ni | Re |
| --- | --- | --- | --- | --- |
| *x* =0.0 | 1.01 | 1.00 | 1.02 | 1.01 |
| *x* = 1.0 | 1.04 | 1.00 | 1.01 | 1.03 |

**(a)**


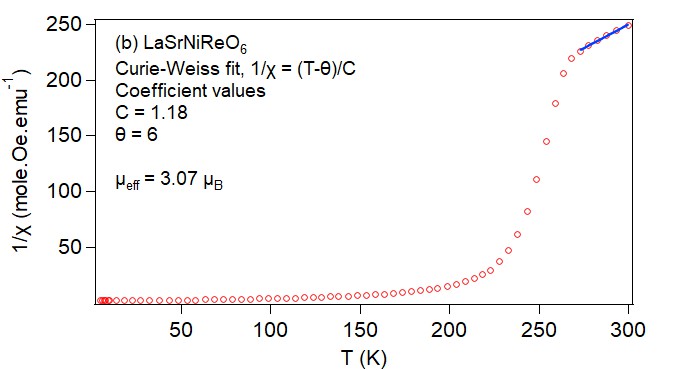

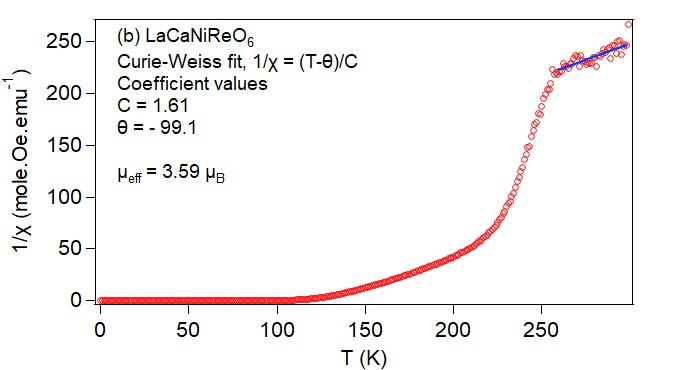


**Figure S2: Curie-Weiss fit:** Curie-Weiss fit for *x* = 0 (a) and *x* = 1 (b). For the spin-only configuration of Ni^2+^ and Re^5+,^ both containing 2 unpaired electrons, the µ_eff_ is expected to be 4 µ_B_. The reduced Re-moment due to the presence of strong spin-orbit coupling results to a reduced µ_eff_. The difference in effective moment between *x* = 0 and *x* = 1 samples could be due to the smaller temperature range of fitting available in our data, where Curie-Weiss regime is not fully achieved. Negative Curie temperatures (θ) in case of *x* = 1 sample indicates antiferro-interaction, while small θ for *x* = 0 sample is an indication of the presence of weak interaction in the system.
